# A comparison of cycloplegic effect of cyclopentolate 0.5% versus 1.0% eye drops with five different refraction measurement modalities in young adults

**DOI:** 10.1007/s00417-024-06658-9

**Published:** 2024-11-23

**Authors:** Klemens Paul Kaiser, Christoph Lwowski, Faisal Nazir, Thomas Kohnen, Yaroslava Wenner

**Affiliations:** Department of Ophthalmology, Goethe-University, Theodor-Stern-Kai 7, 60590 Frankfurt am Main, Germany

**Keywords:** Refraction, Cycloplegia, Retinoscopy, Autorefractometer, Cyclopentolate

## Abstract

**Purpose:**

To compare the refraction before and after cycloplegia with 0.5% and 1.0% cyclopentolate eye drops using five different measurement modalities.

**Methods:**

This prospective, clinical comparative study enrolled 96 eyes of 48 healthy patients with a mean age of 26.6 ± 4.21 years (range: 19–34). Subjective refraction, retinoscopy, and objective refraction were measured using three autorefractometers: Topcon KR-800 (TC), Retinomax K-plus 3 (RM + 3), and Retinomax K-plus Screeen (RM + S) under noncycloplegic and cycloplegic conditions. Cycloplegia was performed in the right eye using 0.5% and in the left eye with 1.0% cyclopentolate eye drops. Differences in refraction in noncycloplegia and cycloplegia, between cycloplegia with 0.5% and 1.0% cyclopentolate, and between the devices were investigated.

**Results:**

Cycloplegic mean spherical equivalent was -1.77 ± 2.34 diopters (D) (-9.75 to + 1.625). All approaches showed a statistically significant hyperopic shift (*p* < 0.001, each) after induction of cycloplegia using both regimes. Lowest median (interquartile range) hyperopic shift was shown by TC (0.25 D (0.38)) and retinoscopy (0.25D (0.75)), and the highest by RM + 3 (0.75 (1.31)). No statistically significant differences between cycloplegia using 0.5% and 1.0% regimens were shown in all modalities (*p* > 0.05, each). In noncycloplegia, there were greater differences compared to cycloplegia. No influence of iris color on the refraction was found.

**Conclusion:**

After induction of cycloplegia all devices showed a hyperopic shift and good comparability to retinoscopy. In all measurement modalities, no significant refraction differences between 0.5% and 1.0% cyclopentolate eye drops were seen. Therefore, 0.5% cyclopentolate was proven to have a sufficient effect with presumably better tolerability.

**Key messages:**

***What is known***

- Cycloplegic refraction is a key test in the evaluation of any patient with active accommodation.
- The most frequently used clinical tests to determine the exact refraction are retinoscopy, subjective refraction, and objective refraction using autorefractometry.

***What is new***

- No significant differences in the refraction between cycloplegia using 0.5% and 1.0% cyclopentolate eye drops were found.
- In noncycloplegia, hand-held autorefractometers tend to measure higher myopia.
- The evaluation of cycloplegic refraction showed good comparability between retinoscopy and subjective refraction as well as three different autorefractometers.

**Supplementary Information:**

The online version contains supplementary material available at 10.1007/s00417-024-06658-9.

## Introduction

Refractive errors in children and young adults are often difficult to diagnose. However, the detection of refractive errors in children is essential to prevent amblyopia, which can have a significant impact on the quality of life [1]. The prevalence of myopia in children is rising sharply worldwide [2]. If it is not detected early and effectively, necessary intervention is not initiated and the myopia progresses [3]. This is followed by complications that permanently impair vision [3]. In children, refraction in cycloplegia is the gold standard due to the wide range of accommodation, but the use of cycloplegia in adults is still controversial [4, 5]. This can lead to ciliary muscle strain with consecutive asthenopia with symptoms such as ocular fatigue, discomfort, and headaches [6]. The most frequently used clinical tests in children to determine the exact refraction are retinoscopy and autorefractometry. As children have a high degree of accommodation, cycloplegic retinoscopy remains the gold standard [7]. Nevertheless, this requires professionally trained staff, the necessary spatial conditions, a corresponding amount of time, and a certain minimum level of cooperation. Given this, several techniques have been developed and are increasingly being used to assess refractive status, as they are much easier to handle and are time-saving [8]. In addition, the increase in people with refractive error has led to a significant increase in demand for refractive surgery, with refraction under cycloplegia being essential in determining manifest refractive error in young adults. [9].

When planning a cycloplegic examination, several considerations must be made, such as which agent to use, optimal dosage, indication for use, and potential side effects [10]. The American Optometric Association's current cycloplegic guidelines recommend cyclopentolate 0.5% in infants and cyclopentolate 1.0% in all children over one year of age [11]. The American Academy of Ophthalmology recommends the use of cyclomydril 0.5% under six months old and cyclopentolate 1.0% over six months old, which are safe and effective [12]. Cyclopentolate is the most commonly used cycloplegic agent with an onset approximately 30 min after the instillation and a duration of action of up to 24 h [10]. Nevertheless, its potential side effects can reach up to grand mal seizure and anticholinergic syndrome, so the lower dosage of cyclopentolate could result in a better tolerability [13].

This study seeks to investigate measurement differences between five techniques that are routinely used in clinical settings to assess refractive error. These include the retinoscopy, subjective refraction, and objective refraction with three different autorefractometers, the first based on proprietary rotary prism technology (Topcon KR-800, Topcon Medical Systems, Inc., Oakland, NJ, USA), the second and third are hand-held autorefractometer (Retinomax K-plus 3 and Retimonax K-plus Screeen), under non-cycloplegic conditions and cycloplegia using two different dosages of cyclopentolate eye drops in a young adolescent population. To date, no comparison of cycloplegia between cyclopentolate eye drops at a concentration of 0.5% and 1.0% has been found in the literature.

## Methods

In this prospective, clinical trial 96 eyes of 48 healthy patients between 19 and 34 years were enrolled at the Department of Ophthalmology, Goethe-University, Frankfurt am Main, Germany. Only patients with clear ocular media were included. Patients with ocular pathologies were excluded, as well as if they were unable to complete the examinations due to other circumstances or if there were contraindications for the induction of cycloplegia using cyclopentolate eye drops. The study protocol was approved by the local ethics committee and the principles of the Declaration of Helsinki were followed. All patients signed an informed consent form.

### Examinations

After all subjects had undergone a basic ophthalmological examination to check for the presence of exclusion criteria, subjective refraction, and objective refraction of both eyes using three different autorefractometers were performed, as well as retinoscopy. The iris color was determined at the slit lamp and divided into "light" (e.g. grey, blue) and "dark" (e.g. brown, green). Pupil size was measured in both eyes before and after induction of cycloplegia using the Retinomax K-plus 3 (Righton, Tokyo, Japan). To induce cycloplegia, preservative-free 0.5% cyclopentolate hydrochloride eye drops (Minims, 0.5% w/v Bausch & Lomb, UK) were applied to the right eye three times at ten-minute intervals. After a waiting period of 20 min after the last drop, the cycloplegia and dilated pupil were finally examined by an experienced ophthalmologist. At the same time, cycloplegia was performed in the left eye using preservative-free 1.0% cyclopentolate hydrochloride eye drops (Minims, 1.0% w/v, Bausch & Lomb, UK).

All examinations and measurements were performed by the two ophthalmologists (F.N. and C.L.) in all subjects enrolled in this study.

#### Subjective Refraction

Conventional subjective refraction (SR) was performed using a standard phoropter. Patients were instructed to focus on the Snellen chart at a distance of five meters while being presented with a series of lenses. Fine-tuning was performed using lenses in 0.25 diopter (D) steps based on the patients' subjective feedback on the improvement in their vision. The aim was to find the maximum positive sphere or the minimum negative sphere with the best visual acuity. The astigmatism was adjusted using the crossed-cylinder technique.

#### Retinoscopy

Retinoscopy was performed by a trained ophthalmologist to detect the spherical and cylindrical components of the refraction. Retinoscopy was performed as the first examination in all patients to be blinded regarding the results of the autorefractometer and to avoid observer bias. The results were additionally checked for accuracy by an experienced ophthalmology consultant (C.L.). RS was performed with a Heine Beta 200 streak retinoscope (Heine Optotechnik GmbH, Herrsching, Germany) and a retinoscopy rack lens set.

#### Autorefractometer

The refractive error, expressed in spherical and cylindrical values, and the astigmatic axis were measured using a Topcon KR-800 (TC) closed-field auto kerato-refractometer (Topcon Medical Systems, Inc., Oakland, NJ, USA).

In addition, each patient was refracted with the Retinomax K-plus 3 (Righton, Tokyo, Japan) (RM + 3). This is a hand-held autorefractometer that is positioned approximately five centimeters in front of one eye according to the manufacturer's instructions. The RM + 3 uses a fogging mechanism to avoid accommodation (source). During the measurement, the patient is asked to focus on an image with a red tulip in the device with the corresponding eye. Keratometry and autorefraction are then performed, whereby eight measurements are evaluated in each case and a representative value is given as a sphere, cylinder, and axis. The range is—18 to + 22 D in 0.25 D steps. The cylindrical range is 12 D.

Finally, all eyes were refracted with the newer hand-held Retinomax K-plus 5 Screen (Righton, Tokyo, Japan) (RM + S) autorefractometer. The refraction was performed concordantly with the autorefraction with the RM + 3. Differences between the RM + 3 and the RM + S include the slightly larger spherical range of—20 to + 23 D in 0.12 and 0.25 D steps of the RM + S. In addition, the RM + S has a so-called "CHILD Mode", whereby acoustic signals and light sources on the device are used to increase attention. The "Focus Assist" allows faster measurements, with different colors on the display indicating the current distance to the eye. If the alignment is correct, the device automatically triggers five measurements, whereby three are automatically repeated if the measurements are unclear.

### Statistical analysis and sample size calculation

Manifest spherical error (MRESph), cylindrical error (MRECyl), and the astigmatic axis, as well as the spherical equivalent (SEQ) were entered into an Excel spreadsheet (version 16.79.1, Microsoft Corporation, Redmond, WA, USA). The SEQ was calculated as: $\text{SEQ}=\text{MRESph}+ \frac{\text{MRECyl}}{2}$. Normal distribution was determined using the Shapiro–Wilk test, whereby all data were not normally distributed (*p* < 0.05). Descriptive statistics were expressed as mean ± standard deviation (SD) if normally distributed, or as median with interquartile range (IQR) if not normally distributed.

Differences between the devices and between cycloplegia and noncycloplegia (0.5% and 1.0%) of MRESph, MRECyl, and SEQ were compared using Wilcoxon tests for paired samples.

The agreement between noncycloplegia and cycloplegia, as well as between cycloplegia using 0.5% and 1.0% cyclopentolate eye drops, and between the measurement methods used was assessed by calculating the bias, defined as the mean difference in the refraction values of the two methods including the upper and lower limits of agreement (LoA) by determining the 95% confidence interval (CI). Agreement between the RS and the other four measurement methods was graphically presented using Bland–Altman plots. In addition, the agreement between the devices was analyzed using inter-class correlation coefficients (ICC). The one-sided Wilcoxon signed rank test was used to determine a statistically significant difference of 0 between the difference of the differences.

To examine differences between iris color (light or dark) and refraction in cycloplegia and noncycloplegia, the Mann–Whitney U-test was used. P-values below 0.05 were considered statistically significant. If necessary, the p-values were corrected according to Bonferroni. SPSS software (version 29.0.1.0; IBM Corporation, Armonk, NC, USA) was used for statistical analysis.

The sample size calculation was performed using the G*Power 3.1 Software (Heinrich Heine University Duesseldorf, Germany). A difference in the measured refraction of 0.5 D can be considered to be clinically relevant. Assuming a standard deviation of 1.0 D, this results in a detectable effect size of 0.5. With a significance level of 0.05 and a second-order error of 0.9, this results in a case number of 47 eyes.

## Results

A total of 96 eyes of 48 patients (35 female) were included in this study. The patients were young adults with a mean age of 26.6 ± 4.16 (range: 19—34) years. Thirteen patients had light irises and 35 had dark irises. In retinoscopy (RS) without induction of cycloplegia, the median (IQR) SEQ was -1.75 (2.19) D (range: -10.50 to + 1.5 D) in the right eye and -1.75 (2.25) D (range: -9.50 to + 1.75 D) in the left eye. The differences between the two eyes were compared using the Wilcoxon test, which showed no statistically significant difference in all modalities (*p* > 0.381 each). After induction of cycloplegia with 0.5% cyclopentolate eye drops in the right eye and 1.0% cyclopentolate eye drops in the left eye, there was a median hyperopic shift of + 0.25 D in both eyes, respectively. Table 1 shows the median (IQR) of the refraction values of the individual methods in both eyes.

**Table 1:** Median and interquartile range (IQR) of noncycloplegia and cycloplegia and all five modalities

Device	Noncycloplegia OD			Cycloplegia 0.5%				Difference		
	MRESph	MRECyl	SEQ	MRESph	MRECyl	SEQ	*p*-value^a^	MRESph	MRECyl	SEQ
**Retinoscopy**	−1.50 (2.63)	−0.50 (1.25)	−1.75 (2.19)	−1.12 (3.00)	−0.50 (1.00)	−1.50 (2.81)	**.003**	0.37 (0.75)	0.00 (0.25)	0.25 (0.75)
**Subjective refraction**	−1.37 (2.50)	−0.50 (4.50)	−1.62 (2.62)	−1.25 (3.19)	−0.50 (1.00)	−1.50 (3.03)	** < .001**	0.25 (1.00)	0.00 (0.00)	0.25 (0.88)
**TOPCON KR-800**	−1.37 (2.69)	−0.75 (0.75)	−1.69 (2.91)	−1.12 (3.00)	−0.50 (0.69)	−1.37 (3.00)	** < .001**	0.25 (0.44)	0.00 (0.25)	0.31 (0.47)
**Retinomax K-plus 3**	−1.62 (2.25)	−0.50 (0.94)	−1.94 (2.31)	−1.00 (2.88)	−0.50 (0.94)	−1.31 (2.78)	** < .001**	0.50 (0.50)	0.00 (0.50)	0.38 (0.63)
**Retinomax K-plus Screeen**	−1.87 (2.69)	−0.75 (1.00)	−2.37 (2.69)	−0.87 (2.75)	−0.50 (0.75)	−1.06 (2.81)	** < .001**	0.75 (1.00)	0.25 (0.25)	0.69 (1.13)
	**Noncycloplegia OS**			**Cycloplegia 1.0%**				**Difference**		
	**MRESph**	**MRECyl**	**SEQ**	**MRESph**	**MRECyl**	**SEQ**	***p*-value**^**a**^	**MRESph**	**MRECyl**	**SEQ**
**Retinoscopy**	−1.25 (2.69)	−0.50 (1.19)	−1.75 (2.25)	−1.00 (2.75)	−0.50 (0.75)	−1.44 (2.59)	**.017**	0.25 (0.94)	0.00 (0.25)	0.25 (0.87)
**Subjective refraction**	−1.50 (2.69)	−0.50 (1.00)	−1.69 (2.72)	−1.37 (2.94)	−0.37 (0.94)	−1.62 (2.84)	** < .001**	0.50 (0.50)	0.00 (0.00)	0.37 (0.50)
**TOPCON KR-800**	−1.50 (3.00)	−0.50 (1.00)	−1.75 (2.50)	−1.12 (2.94)	−0.50 (1.00)	−1.56 (2.78)	** < .001**	0.25 (0.44)	0.00 (0.25)	0.25 (0.37)
**Retinomax K-plus 3**	−1.37 (2.50)	−0.50 (0.94)	−1.81 (2.47)	−0.87 (3.00)	−0.25 (0.75)	−1.31 (2.75)	** < .001**	0.50 (0.50)	0.00 (0.50)	0.50 (0.50)
**Retinomax K-plus Screeen**	−1.75 (2.94)	−0.50 (1.00)	−1.94 (3.12)	−1.00 (2.94)	−0.50 (1.00)	−1.25 (3.09)	** < .001**	0.75 (1.19)	0.00 (0.50)	0.75 (1.31)

^*a*^*p*-value was calculated using Mann–Whitney U-test

*MRESph* manifest spherical error*, MRECyl* manifest cylindrical error*, SEQ* spherical equivalent*, OD* right eye*, OS* left eye

In noncycloplegia and cycloplegia as well there was good comparability between the refraction of the right and left eyes with no statistically significant differences (*p* > 0.05 each).

The pupil diameter of the right eye in noncycloplegia was on average 4.96 ± 0.61 mm (Range: 3.60 – 6.60 mm), and in the left eye 4.98 ± 0.60 mm (Range: 3.60 – 6.80 mm) and in cycloplegia 7.74 ± 0.67 (Range: 6.00 – 8.80 mm) and 7.84 ± 0.61 mm (Range: 6.60 – 8.80 mm), respectively. In both eyes, the change in pupil size before and after induction of cycloplegia was statistically significant (each < 0.001). While the mean pupil size in noncycloplegia did not differ significantly (*p* = 0.345), there was a statistically significant (difference: 0.10 ± 0.21 mm, *p* = 0.003) larger mean pupil diameter of the left eye (1.0% regimen).

### Noncycloplegia versus cycloplegia

In all devices, a statistically significant hyperopic shift, regarding the SEQ, was detected after induction of cycloplegia, with the extent of the hyperopic shift being most prominent in both groups with the RM + 3 and RM + S. The refraction values of the five different approaches with and without cycloplegia, shown as MRESph, MRECyl, and SEQ, as well as the difference with p-values, are presented in Table 1.

Looking at MRESph, all devices showed a statistically significant hyperopic shift (*p* < 0.001 each). The lowest hyperopic shift was shown by retinoscopy (0.25 D) and the highest value by RM + S (0.69 and 0.75 D, right and left eye, respectively). Looking at the MRECyl, there was only a minor change in all groups, with a statistically significant change only in the retinoscopy (0.00 (0.25) D, *p* = 0.011) and (RM + S 0.00 (0.50) D, *p* = 0.001) groups with cycloplegia 0.5%. The one-sided t-test showed a statistically significant deviation of the differences between noncycloplegia and cycloplegia (0.5% and 1.0%) from 0 (*p* < 0.005 each) in all devices.

A subgroup analysis was performed to show differences between hyperopic and myopic eyes. The median (IQR) of the difference in SEQ between noncycloplegia and cycloplegia of both eyes are shown in Table 2.

**Table 2:** Median and interquartile range (IQR) of the cycloplegic and noncycloplegic spherical equivalent (SEQ) as well as the differences in SEQ of the right (0.5% cyclopentolate) and the left eye (1.0% cyclopentolate) of all five modalities divided into hyperopic (*n* = 12) and myopic (*n* = 36) eyes

**Hyperopes ≥ 0 D (*n* = 12)**						
Device	**Noncyclo. OD**	**Cyclo. 0.5%**	**Difference**	**Noncyclo. OS**	**Cyclo. 1.0%**	**Difference**
Retinoscopy	0.00 (1.34)	0.50 (0.62)	0.50 (1.19)	−0.37 (0.91)	0.50 (0.97)	0.44 (0.87)
Subjective refraction	−0.44 (0.59)	0.44 (0.72)	0.87 (0.87)	−0.50 (0.69)	0.31 (0.84)	0.69 (0.66)
TOPCON KR-800	−0.12 (0.72)	0.37 (0.69)	0.56 (0.50)	−0.25 (0.69)	0.44 (0.97)	0.50 (0.66)
Retinomax K-plus 3	−0.62 (1.00)	0.44 (0.78)	1.06 (1.09)	−0.56 (1.12)	0.50 (0.69)	0.69 (1.06)
Retinomax K-plus Screeen	−0.87 (1.16)	0.37 (0.56)	1.06 (1.69)	−0.69 (1.12)	0.19 (0.91)	0.75 (1.40)
**Myopes < 0 D (*n* = 36)**						
Device	**Noncyclo. OD**	**Cyclo. 0.5%**	**Difference**	**Noncyclo. OS**	**Cyclo. 1.0%**	**Difference**
Retinoscopy	−2.00 (2.69)	−2.25 (2.69)	0.25 (0.72)	−2.00 (2.60)	−2.12 (3.12)	0.06 (0.81)
Subjective refraction	−2.44 (2.78)	−2.19 (2.69)	0.25 (0.50)	−2.69 (3.00)	−2.12 (2.66)	0.25 (0.50)
TOPCON KR-800	−2.44 (2.47)	−2.06 (2.50)	0.25 (0.37)	−2.37 (2.87)	−2.25 (2.78)	0.25 (0.25)
Retinomax K-plus 3	−2.44 (2.34)	−1.94 (2.47)	0.37 (0.62)	−2.37 (3.16)	−2.25 (2.66)	0.50 (0.59)
Retinomax K-plus Screeen	−2.87 (3.03)	−2.12 (2.84)	0.62 (1.00)	−2.75 (3.22)	−2.25 (3.19)	0.69 (0.81)

*Cyclo* cycloplegia, *Noncyclo* noncycloplegia, *OD* right eye, *OS* left eye*, SEQ* spherical equivalent

### Changes in refraction after cycloplegia 0.5% versus 1.0%

To investigate differences between the two cycloplegic regimes, the differences in refraction between noncycloplegic and cycloplegic were calculated and compared between the two groups. The SEQ showed a median difference of both eyes of 0.25 D in the retinoscopy and TC, 0.31 D in the SR, 0.50 D in the RM + 3, and 0.75 D in the RM + S groups. The median differences and the IQR between the 0.5% and 1.0% regimes are shown as MRESph, MRECyl, and SEQ in Table 3. Using the one-sided-t-test, no statistically significant deviation from 0 (> 0.05 in each case) was found for all approaches, as well as after subgroup analysis when only hyperopic eyes (> 0 D) and myopic eyes (< 0 D) were studied.

**Table 3:** Median and interquartile range (IQR) of the difference between the differences of the cycloplegia 0.5% and 1.0% regime of the five modalities

Device	MRESph	MRECyl	SEQ
Retinoscopy	0.00 (0.50)	0.00 (0.25)	0.00 (0.50)
Subjective refraction	0.00 (0.50)	0.00 (0.25)	0.00 (0.44)
TOPCON KR-800	0.00 (0.25)	0.00 (0.25)	0.00 (0.38)
Retinomax K-plus 3	0.00 (0.50)	0.00 (0.50)	0.00 (0.53)
Retinomax K-plus Screeen	0.13 (1.00)	0.25 (0.75)	0.13 (0.88)

*MRESph* manifest spherical error*, MRECyl* manifest cylindrical error*, SEQ* spherical equivalent

### Differences between the devices

When looking at the differences between the devices in noncycloplegia, there was a statistically significant difference between almost all devices in both eyes, except for retinoscopy vs. TC (*p* = 0.702 and *p* = 0.132; 0.5% and 1.0% group, respectively), and between SR vs. RM + 3 (*p* = 0.896 and *p* = 0.943; 0.5% and 1.0% group, respectively).

**Fig. 1:**
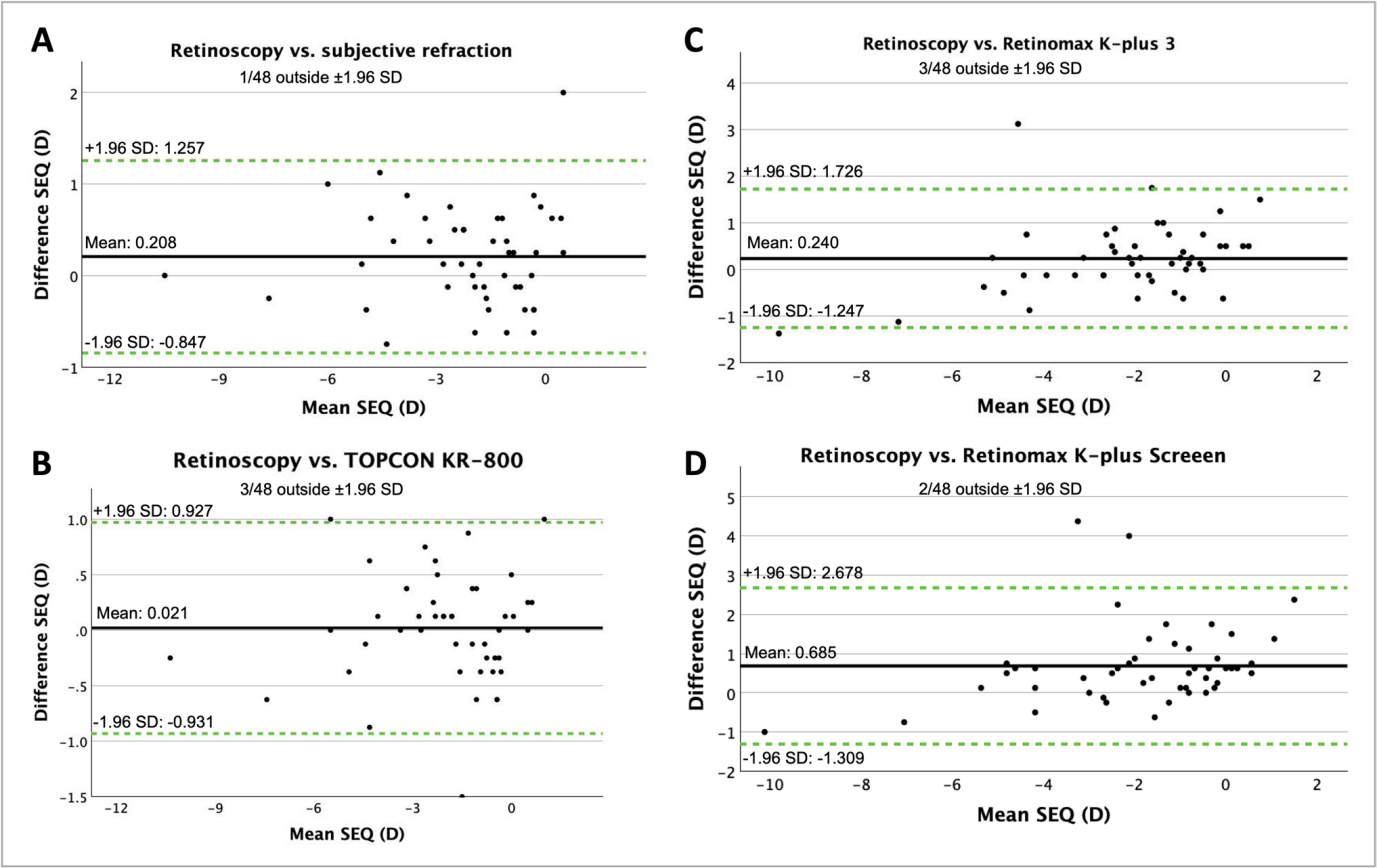
Bland–Altman plots show the difference in spherical equivalent (SEQ) in diopters (D) in noncycloplegia between retinoscopy and subjective refraction (**A**), retinoscopy and TOPCON KR-800 (**B**), retinoscopy and Retinomax K-plus 3 (**C**) and retinoscopy and Retinomax K-plus Screeen (**D**). The middle line shows the mean difference between the two modalities, and the two green dashed lines show the 95% limits of agreement

**Fig. 2:**
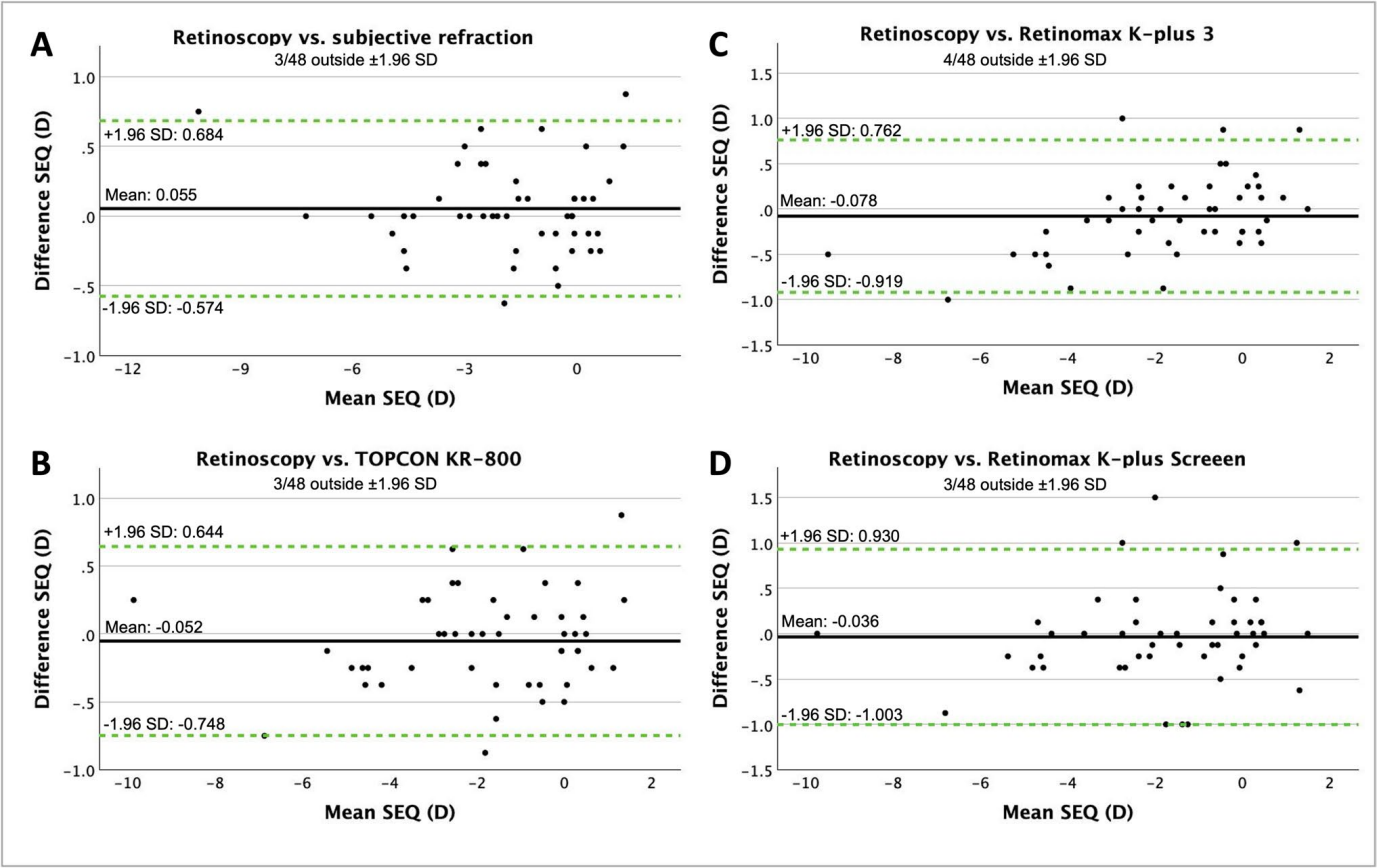
Bland–Altman plots show the difference in spherical equivalent (SEQ) in diopters (D) in cycloplegia with the 0.5% regime between retinoscopy and subjective refraction (**A**), retinoscopy and TOPCON KR-800 (**B**), retinoscopy and Retinomax K-plus 3 (**C**) and retinoscopy and Retinomax K-plus Screeen (**D**). The middle line shows the mean difference between the two modalities, and the two green dashed lines show the 95% limits of agreement

**Fig. 3:**
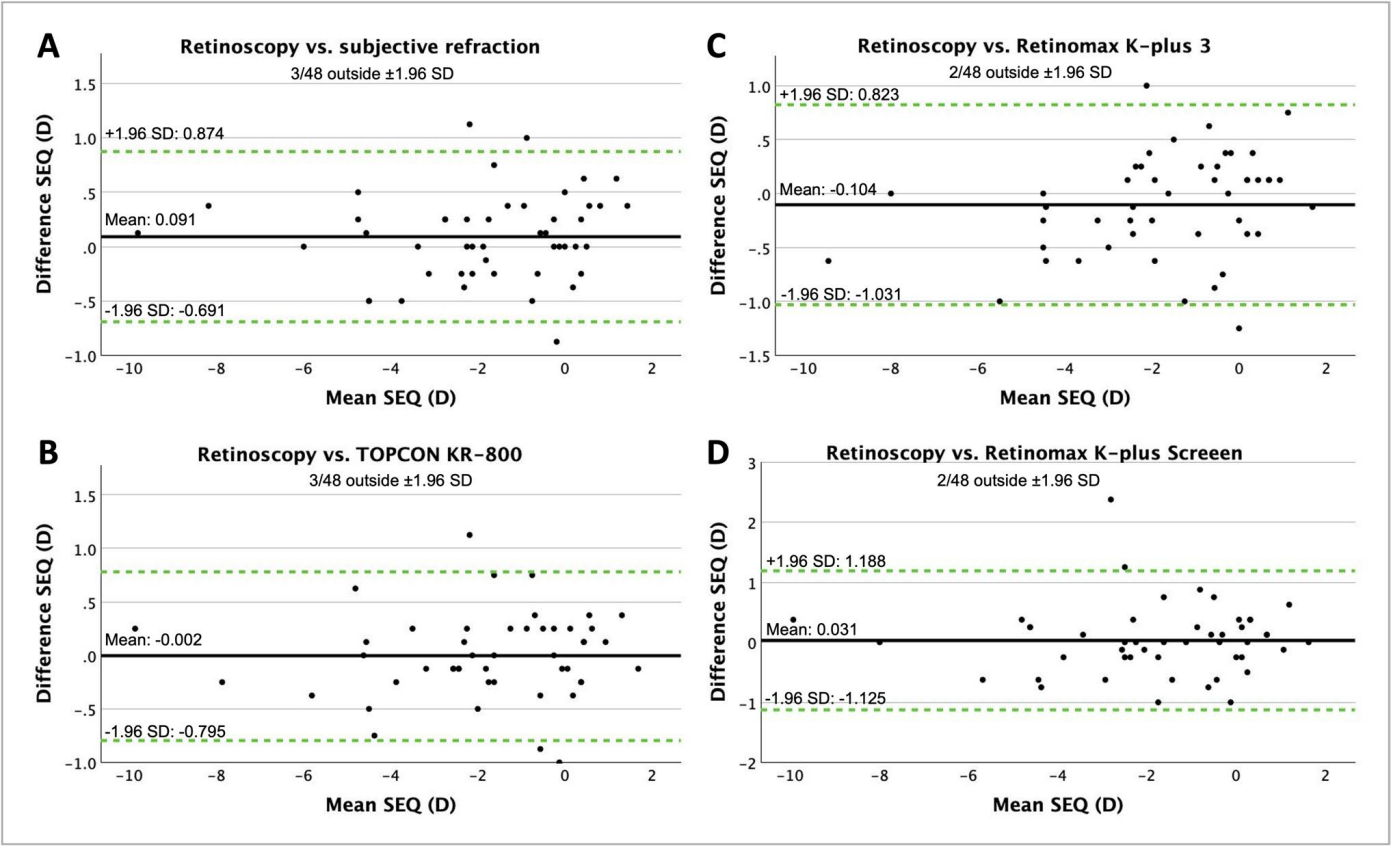
Bland–Altman plots show the difference in spherical equivalent (SEQ) in diopters (D) in cycloplegia with the 1.0% regime between retinoscopy and subjective refraction (**A**), retinoscopy and TOPCON KR-800 (**B**), retinoscopy and Retinomax K-plus 3 (**C**) and retinoscopy and Retinomax K-plus Screeen (**D**). The middle line shows the mean difference between the two modalities, and the two green dashed lines show the 95% limits of agreement

After cycloplegia according to the 0.5% regimen, there was better agreement between the devices, with only a statistically significant difference between the SR vs. TC (*p* = 0.002) and SR vs. RM + 3 (*p* = 0.015). In contrast to the noncycloplegia, where a statistically significant difference to RM + S was found in all devices, no statistically significant difference was found in the cycloplegia 0.5% group. Similar results were also observed after induction of cycloplegia following the 1.0% regimen, with likewise statistically significant differences between SR vs. TC (*p* = 0.005) and SR vs. RM + 3 (*p* < 0.001). Additionally, a statistically significant difference in the direct comparison of RM + 3 vs. RM + S was found in this group (*p* = 0.012). Bland–Altman plots were created between the retinoscopy group and the other approaches showing the mean difference and the 95% limit of agreement in noncycloplegia (Fig. 1A-D), cycloplegia with the 0.5% regime (Fig. 2A-D), and cycloplegia with the 1.0% regime (Fig. 3A-D).

The p-values of the direct comparison between the different devices are listed in Table 4. The inter-class correlation coefficients (ICC) between the different devices in noncycloplegic and the two different cycloplegic conditions are listed in Table 5. The highest ICC values (> 0.97) were found between retinoscopy and SR and retinoscopy and TC, both in noncycloplegia and cycloplegia. The median (IQR) of the differences in SEQ between the devices of both the right and left eye are presented in supplementary Table 1.

**Table 4:** *P*-values for the comparison of the manifest refraction of the modalities in non-cycloplegia and cycloplegia. Statistically significant p-values are written in bold

Device	Subjective refraction	TOPCON KR-800	Retinomax K-plus 3	Retinomax K-plus Screeen
**Noncycloplegia OD**				
Retinoscopy	**0.013**	0.702	**0.03**	** < .001**
Subjective refraction	−	**0.002**	0.896	**0.002**
TOPCON KR-800	−	−	**0.027**	** < .001**
Retinomax K-plus 3	−	−	−	** < .001**
**Noncycloplegia OS**				
Retinoscopy	**0.002**	0.132	**0.003**	** < .001**
Subjective refraction	−	**0.001**	0.943	**0.01**
TOPCON KR-800	−	−	**0.01**	** < .001**
Retinomax K-plus 3	−	−	−	** < .001**
**Cycloplegia 0.5% OD**				
Retinoscopy	0.347	0.273	0.126	0.343
Subjective refraction	−	**0.002**	**0.015**	0.097
TOPCON KR-800	−	−	0.374	0.927
Retinomax K-plus 3	−	−	−	0.419
**Cycloplegia 1.0% OS**				
Retinoscopy	0.159	> 0.999	0.16	0.935
Subjective refraction	−	**0.005**	** < .001**	0.059
TOPCON KR-800	−	−	0.059	0.901
Retinomax K-plus 3	−	−	−	**0.012**

*p*-values were calculated using Mann–Whitney U-test

*RS* retinoscopy*, SR* subjective refraction, *TC* TOPCON KR-800*, RM* + *3* Retinomax K-plus 3*, RM* + *S* Retinomax K-plus Screeen*, OD* right eye*, OS* left eye

**Table 5:** Inter-class correlation coefficient between the devices in noncycloplegia and cycloplegia

Device	Subjective refraction	TOPCON KR-800	Retinomax K-plus 3	Retinomax K-plus Screeen
**Noncycloplegia OD**				
Retinoscopy	0.988	0.983	0.963	0.919
Subjective refraction	−	0.980	0.944	0.877
TOPCON KR-800	−	−	0.946	0.868
Retinomax K-plus 3	−	−	−	0.915
**Noncycloplegia OS**				
Retinoscopy	0.972	0.983	0.963	0.890
Subjective refraction	−	0.989	0.974	0.916
TOPCON KR-800	−	−	0.980	0.915
Retinomax K-plus 3	−	−	−	0.940
**Cycloplegia 0.5% OD**				
Retinoscopy	0.995	0.994	0.928	0.988
Subjective refraction	−	0.995	0.870	0.982
TOPCON KR-800	−	−	0.893	0.988
Retinomax K-plus 3	−	−	−	0.891
**Cycloplegia 1.0% OS**				
Retinoscopy	0.993	0.993	0.989	0.985
Subjective refraction	−	0.995	0.986	0.986
TOPCON KR-800	−	−	0.989	0.988
Retinomax K-plus 3	−	−	−	0.986

*OD* right eye, *OS* left eye

### Differences depending on the iris color

A subgroup analysis was performed to determine whether differences can be detected after induction of cycloplegia in eyes with less pigmented and highly pigmented irises. The differences in SEQ before and after induction of cycloplegia were compared depending on iris color (light or dark). No statistically significant difference was found in the 0.5% group and 1.0% group, as well as for the different devices in the Mann–Whitney U test for independent samples (*p* > 0.05 in each case).

## Discussion

In recent decades, autorefractometers have become increasingly important for vision screening, and clinical practice, but also for scientific purposes to determine objective refraction in children as well as adults [7–11]. Some studies have already described a more pronounced difference between cycloplegic and noncycloplegic refraction in children and adults, which is particularly due to the higher accommodative capacity [14–18]. Since cycloplegic refraction takes more time and is associated with visual impairment that lasts for several hours, the aim is to obtain the best possible assessment of noncycloplegic refraction with minimal accommodation [10]. We conducted this study to assess differences between the different devices in noncycloplegia, as well as under cycloplegic conditions in young adults using 0.5% and 1.0% cyclopentolate eye drops.

### Noncycloplegia versus cycloplegia

In our study, all approaches showed a hyperopic shift after initiation of cycloplegia as seen in Table 1, considerably more prominent in hyperopic eyes compared to myopic eyes (Table 2). The median difference was least pronounced in the retinoscopy and SR with 0.25 D. The greatest effect of cycloplegia was seen with hand-held ARs (RM + 3 and especially RM + S). The results of hyperopic shift after induction of cycloplegia are generally consistent with most previous studies. The Tehran Eye study showed a mean difference in SEQ using an autorefractometer (Topcon KR-8000, Topcon, Tokyo, Japan) of around 0.4 D in the 16 to 20 age group. This level was maintained until the age group of 46 to 50 years before it decreased to about 0.2 D [19]. The AUSES study with 7971 participants from a school in China with a mean age of 20.2 years also showed a mean difference of 0.83 ± 0.81 D between noncycloplegia and cycloplegia using an autorefractor (HUVITZ HRK-7000A; Huvitz, Gunpo, South Korea) [20]. In the myopic subgroup, the difference was 0.69 ± 0.69 D [18]. Therefore, if the prescription of spectacles is based on non-cycloplegic refraction, this could lead to minus over-correction, resulting in increased accommodative efforts and asthenopic discomfort.

The hyperopic refractive change caused by cycloplegic eye drops is primarily the result of accommodation paralysis. Cycloplegics lead to relaxation of the ciliary muscle, which keeps the zonular fibers under tension, resulting in a flattening of the lens curvature and thus a reduction in refractive power. Some studies have investigated the changes in ocular biological parameters after cycloplegia [21–26]. In a study of 2′049 patients with a mean age of 8.99 ± 3.23 years (range: 1 to 21 years), there was a significant increase in central corneal thickness and anterior chamber depth after cycloplegia using both 1% atropine and 0.5% phenylephrine eye drops, but no significant change in this overall population with regard to axial length, keratometry values and horizontal corneal diameter (each *p* > 0.05) [21]. Therefore, the hyperopic shift is not essentially due to a shortening of the axial length after the induction of cycloplegia.

### Changes in refraction after cycloplegia 0.5% vs. 1.0%

We found no statistically significant differences between the use of 0.5% and 1.0% cyclopentolate eye drops across all measurement modalities (*p* > 0.05 in each case). While all devices except RM + S demonstrated a median difference of 0.00 D between the two regimens, the median difference between the 0.5% and 1.0% eye drops with RM + S, exhibiting a median SEQ difference of 0.13 D towards higher myopic values with 1.0% drops (Table 3). Given this result is counterintuitive, we hypothesize that it was a statistical coincidence. Looking at the MRECyl results before and after induction of cycloplegia, a median difference of ≤ 0.25 D was found in both eyes for each device. It is known that the cycloplegia hardly changes the MRECyl [27]. MRECyl showed with RM + S a median difference of 0.25 D. This could be related with a larger pupil size in cycloplegia with 1.0% compared to 0.5%. Prabakaran et al. showed that astigmatism measured in cycloplegic refraction (1% cyclopentolate) with the hand-held autorefractor (Retinomax K-Plus 2) and table-mounted autorefractor (Canon FK-1) was significantly greater than that measured with retinoscopy [28]. Nevertheless, the discrepancy is of a magnitude that is clinically inconsequential. To date, no comparison of refraction in cycloplegia between cyclopentolate eye drops at a concentration of 0.5% and 1.0% has been found in the literature. Looking at possible side effects of cyclopentolate eye drops, ocular side effects like increased pressure, potential corneal damage or cloudy vision, and systemic side effects including fever, tachycardia, convulsions, and delirium were described [10, 29, 30]. One case report documented a case of a 15-month-old child with cyclopentolate-induced delirium [29]. Another case report revealed toxicity in a 6-year-old child after three drops of cyclopentolate 1% developing mydriasis, incoherent speech, visual hallucinations, and dry mouth [30]. Our study showed very comparable results between 0.5% and 1.0% cyclopentolate eye drops. Therefore, in our opinion, in most cases, the use of the lower concentrated eye drops is sufficient and could contribute to reduction of possible side effects.

### Differences between the devices

By using visual targets in the distance, such as in subjective refraction, accommodation can be reduced compared to closed-field refraction systems [15]. Studies have shown that closed-field AR systems tend to deliver more myopic results, which has led to the development of open-field refraction systems, for example [15]. Fogging mechanisms, as used in TC, RM + 3, and RM + S, are another technical option for reducing accommodation. However, authors have already discussed that the use of the fogging mechanism as an accommodative control mechanism could be insufficient, as other factors also play a role in accommodation and may not be neutralized with fogging [15]. Our results showed a myopic bias in noncycloplegia, particularly in RM + S (supplementary Table 1). The median difference between RM + S and retinoscopy in noncycloplegia was -0.56 and -0.38 D (right and left eye, respectively; each *p* < 0.001). Less pronounced differences were found when comparing RM + 3 and retinoscopy in non-cycloplegia (-0.25 for both eyes), but also with statistically significant differences in both eyes (*p* < 0.05 each). While the differences with the handheld ARs were more pronounced, interestingly, our study showed good comparability between the retinoscopy and the table-mounted TC with no statistically significant difference (*p* = 0.702 and *p* = 0.132; right and left eye, respectively). The median difference between the two modalities was -0.06 and 0.00 D (right and left eye). Based on these findings, we would rather recommend to use TC for autorefraction in non-cycloplegic condition than RM + 3 and RM + S.

Our study showed good comparability between retinoscopy, TC, RM + 3, and RM + S under cycloplegic conditions with a median difference of ≤ 0.13 D each (supplementary Table 1) and an ICC ≥ 0.89 and ≥ 0.98 for 0.5% and 1% regimen (Table 5). A retrospective study investigated the comparability between the hand-held RM + 3 and the table-mounted TC in a pediatric population (7.1 ± 2.2 years, range: 3 to 16 years) in cycloplegic and non-cycloplegic conditions. While virtually identical data were found in non-cycloplegia, a hyperopic bias of + 0.50 D measured with the RM + 3 was found in cycloplegia [31]. In our study, a myopic bias of approximately -0.13 D was measured with the RM + 3 compared to the TC in non-cycloplegic conditions, while a hyperopic bias of + 0.00 D and + 0.13 D (0.5% and 1.0% regimen, respectively) was measured in cycloplegia. Another study with 200 patients aged between 4 and 12 years examined the comparability between the RM + 3 and the retinoscopy in cycloplegic conditions, describing good comparability without significant differences [32]. While our results in non-cycloplegia showed statistically significant differences between the RM + 3 and retinoscopy (*p* = 0.03 and *p* = 0.003; right and left eye, respectively), there were also no differences after induction of cycloplegia (*p* = 0.126 and *p* = 0.16; 0.5% and 1.0% regimen, respectively). A similar picture emerges in our study when comparing RM + S with other modalities. While under noncycloplegic conditions a statistically significant difference to retinoscopy, SR, TC, and RM + 3 could be found (each *p* ≤ 0.01), the comparability in cycloplegia was significantly better except for the comparison to RM + 3 after the 1.0% regime (*p* = 0.012). Therefore, we conclude that TC, RM + 3 and RM + S are equally suitable to measure automatic refraction in cycloplegia in young adults with myopia and low hyperopia.

Statistically significant differences were found when comparing the SR to the other devices (Table 4). For non-cycloplegia, only the comparison with the RM + 3 showed no statistically significant difference (*p* = 0.896 and *p* = 0.943; right and left eye, respectively). There was a statistically significant difference compared to all other devices with the median difference < 0.2 D (supplementary Table 1). For cycloplegia, there were statistically significant differences between SR and TC (*p* = 0.002 and *p* = 0.005, respectively) and RM + 3 (*p* = 0.015 and *p* < 0.001, respectively) in both the 0.5% and 1.0% regimens. The median difference was ≤ 0.25 D which was similar to non-cycloplegia and likely attributable to a higher effect of SR on accommodation [17]. Similar findings were reported by Yuexin et al. who investigated the influence of accommodative function on the difference between cycloplegic and noncycloplegic subjective and automated refraction using either a TOPCON KR-8100 or KR-8800 (Topcon Medical System, Japan) in 3268 individuals with a mean age of 27.3 ± 6.9 years. The results showed that under cycloplegic conditions, the SR provided approximately -0.3 D more myopic results than AR [17]. These results highlight that refraction values cannot be solely based on AR in cycloplegia, irrespective of the modality used. Prior to undergoing refractive surgery or receiving a glasses prescription, patients require a subjective refraction in cycloplegia to ensure optimal visual comfort.

### Differences in pupil size

The results of our study indicated a statistically significant difference in pupil size between the two regimens. While there was no statistically significant difference between the two eyes in noncycloplegia (*p* = 0.345), there was a statistically significant larger pupil after induction of cycloplegia with 1% cyclopentolate eye drops compared to the 0.5% regimen (*p* = 0.003). The mean difference was 0.10 mm, which did not influence most refraction values in both eyes. The somewhat higher cylinder measured with RM + S in the 1% regimen could have been influenced by a larger pupil size [28]. However, it is possible that the difference in pupil size between children and older adults in the two regimens could have been different.

## Conclusion and study limitations

The strengths of the present study include in particular the prospective setting and the five different modalities investigated to determine refraction. Another strength is that in our study, cycloplegia was induced in the right eye with 0.5% cyclopentolate eye drops and in the left eye with 1.0% cyclopentolate eye drops. All examinations in this study were performed by the same examiner, with a second examiner checking the accuracy of the refraction results in the retinoscopy. Since retinoscopy was performed first in all participants, observer bias was minimized.

The study has some limitations, in particular, that most of the patients were young adults with myopia. Therefore, no reliable results can be provided for children of young age. We did not include children in this study, as subjective refraction in this cohort is partly impossible due to reduced cooperation and side effects of cyclopentolate are higher [29, 30]. As a compromise, we included young adults. As there were no patients with high hyperopia in our study population, the hyperopic shift in cycloplegic conditions could be underestimated. Moreover, this study did not examine the subjective effect of cycloplegia associated with the two regimens, precluding the presentation of findings pertaining to photophobia and reading ability.

To conclude, a hyperopic shift was seen in all measurement modalities after induction of cycloplegia without significant differences between the use of 0.5% and 1.0% cyclopentolate eye drops. While greater differences were found in non-cycloplegia, all devices showed good comparability with retinoscopy after the introduction of cycloplegia. In conclusion, the sufficient cycloplegic effect of 0.5% cyclopentolate eye drops in comparison to 1.0% cyclopentolate was proven with presumably better tolerability with lower side effects.

## Supplementary Information

Below is the link to the electronic supplementary material.

Supplementary file1 (DOCX 15 KB)

## Data Availability

All data generated or analyzed during this study are included in this article. Further enquiries can be directed to the corresponding author.
