# Supplementary material for: A comparison of cycloplegic effect of cyclopentolate 0.5% versus 1.0% eye drops with five different refraction measurement modalities in young adults
Source: Graefes Arch Clin Exp Ophthalmol. 2024 Nov 23;263(4):1135–46. doi: 10.1007/s00417-024-06658-9 (PMC12095448; doi:10.1007/s00417-024-06658-9)
Supplement: Supplementary file 1 — Supplementary file1 (DOCX 15 KB) [file 417_2024_6658_MOESM1_ESM.docx]

| **Supplementary Table 1:** The interdevice differences of the spherical equivalent are expressed as the median (interquartile range). | | | | | |
| --- | --- | --- | --- | --- | --- |
| Device | Subjective refraction | TOPCON KR-800 | Retinomax K-plus 3 | Retinomax K-plus Screeen |  |
| **Noncycloplegia OD** | | | | | |
| Retinoscopy | 0.19 (0.75) | 0.06 (0.53) | 0.25 (0.63) | 0.56 (0.75) |  |
| Subjective refraction | - | -0.13 (0.41) | 0.13 (0.75) | 0.19 (0.91) |  |
| TOPCON KR-800 | - | - | 0.13 (0.53) | 0.44 (0.91) |  |
| Retinomax K-plus 3 | - | - | - | -0.38 (0.63) |  |
| **Noncycloplegia OS** | | | | | |
| Retinoscopy | 0.13 (0.53) | 0.00 (0.53) | 0.25 (0.63) | 0.38 (1.00) |  |
| Subjective refraction | - | -0.06 (0.25) | 0.00 (0.66) | 0.19 (1.03) |  |
| TOPCON KR-800 | - | - | 0.06 (0.50) | 0.25 (0.91) |  |
| Retinomax K-plus 3 | - | - | - | 0.19 (0.75) |  |
| **Cycloplegia 0.5% OD** | | | | | |
| Retinoscopy | 0.00 (0.28) | 0.00 (0.41) | 0.00 (0.25) | 0.00 (0.38) |  |
| Subjective refraction | - | 0.00 (0.25) | -0.13 (0.28) | -0.06 (0.38) |  |
| TOPCON KR-800 | - | - | 0.00 (0.38) | 0.00 (0.25) |  |
| Retinomax K-plus 3 | - | - | - | 0.00 (0.19) |  |
| **Cycloplegia 1.0% OS** | | | | | |
| Retinoscopy | 0.06 (0.63) | 0.00 (0.50) | 0.00 (0.53) | 0.00 (0.54) |  |
| Subjective refraction | - | -0.13 (0.50) | -0.25 (0.38) | -0.13 (0.50) |  |
| TOPCON KR-800 | - | - | -0.13 (0.28) | 0.00 (0.38) |  |
| Retinomax K-plus 3 | - | - | - | 0.13 (0.28) |  |

*OD = right eye; OS = left eye*
